# Characteristics of female breast cancer in japan: annual report of the National Clinical Database in 2018

**DOI:** 10.1007/s12282-022-01423-4

**Published:** 2022-12-22

**Authors:** Keiichiro Tada, Hiraku Kumamaru, Hiroaki Miyata, Sota Asaga, Kotaro Iijima, Etsuyo Ogo, Takayuki Kadoya, Makoto Kubo, Yasuyuki Kojima, Kenta Tanakura, Kenji Tamura, Masayuki Nagahashi, Naoki Niikura, Naoki Hayashi, Minoru Miyashita, Masayuki Yoshida, Shinji Ohno, Shigeru Imoto, Hiromitsu Jinno

**Affiliations:** 1 Department of Breast and Endocrine Surgery, Nihon University School of Medicine, 30-1 Oyaguchikamicho, Itabashi-Ku, Tokyo, 173-8610 Japan; 2 Department of Healthcare Quality Assessment, University of Tokyo, 7-3-1 Hongo, Bunkyo-Ku, Tokyo, 113-8655 Japan; 3 Department of Breast Surgery, Kyorin University School of Medicine, 6-20-2 Shinkawa, Mitaka, Tokyo 181-8611 Japan; 4 Department of Breast Oncology, Juntendo University, 3-1-3 Hongo, Bunkyo-Ku, Tokyo, 113-8431 Japan; 5 Department of Radiology, Kurume University School of Medicine, 67 Asahi-Machi, Kurume, Fukuoka 830-0011 Japan; 6 Department of Surgical Oncology, Research Institute for Radiation Biology and Medicine, Hiroshima University, 1-2-3 Kasumi, Minami-Ku, Hiroshima, 734-8553 Japan; 7 Department of Surgery and Oncology, Graduate School of Medical Sciences, Kyushu University, 3-1-1 Maidashi Higashi-Ku, Fukuoka, 812-8582 Japan; 8 Division of Breast and Endocrine Surgery, Department of Surgery, St. Marianna University School of Medicine, 2-16-1 Sugao, Miyamae-Ku, Kawasaki, 216-8511 Japan; 9 Plastic and Reconstructive Surgery, Mitsui Memorial Hospital, 1 Kanda-Izumicho, Chiyoda-Ku, Tokyo, 101-8643 Japan; 10 Department of Medical Oncology, Shimane University Hospital, 89-1 Enya-Cho, Izumo-Shi, Shimane, 693-8501 Japan; 11 Department of Surgery, Division of Breast and Endocrine Surgery, School of Medicine, Hyogo Medical University, 1-1 Mukogawa, Nishinomiya, Hyogo 663-8501 Japan; 12 Department of Breast Oncology, Tokai University School of Medicine, 143 Shimokasuya, Isehara, Kanagawa 259-1193 Japan; 13 Department of Breast Surgical Oncology, St. Luke’s International Hospital, 9-1 Akashicho, Chuo-Ku, Tokyo, 104-8560 Japan; 14 Department of Breast and Endocrine Surgical Oncology, Tohoku University School of Medicine, Seiryo-Machi, Aoba-Ku, Sendai, 980-8574 Japan; 15 Department of Diagnostic Pathology, National Cancer Center Hospital, 5-1-1 Tsukiji, Chuo-Ku, Tokyo, 104-0045 Japan; 16 Breast Oncology Center, Cancer Institute Hospital, 3-8-31 Ariake, Koutou-Ku, Tokyo, 135-8550 Japan; 17 Department of Surgery, Teikyo University School of Medicine, 2-11-1 Kaga, Itabashi-Ku, Tokyo, 173-8606 Japan

**Keywords:** Japanese Breast Cancer Society, Breast neoplasms, Registry, National Clinical Database in Japan, Annual report

## Abstract

Information regarding patients who were treated for breast cancer in 2018 was extracted from the National Clinical Database (NCD), which is run by Japanese physicians. This database continues from 1975, created by the Japanese Breast Cancer Society (JBCS). A total of 95,620 breast cancer cases were registered. The demographics, clinical characteristics, pathology, surgical treatment, adjuvant chemotherapy, adjuvant endocrine therapy, and radiation therapy of Japanese breast cancer patients were summarized. We made comparisons with other reports to reveal the characteristics of our database. We also described some features in Japanese breast cancer that changed over time. The unique characteristics of breast cancer patients in Japan may provide guidance for future research and improvement in healthcare services.

## Introduction

There are three breast cancer registries in Japan. The first is a national registry for which data are gathered through local governments [1]. These data are provided by all Japanese hospitals and some clinics. The second is a registry for which data are collected by cancer hospitals designated by Japanese authorities [2]. The third is a registry run by the Japanese Breast Cancer Society (JBCS), which consists of experts specializing in breast cancer. Therefore, the last registry contains a wide range of breast cancer information. This registry was integrated into the National Clinical Database (NCD) in 2012. The NCD, a Japanese online database run by medical experts, mainly contains data regarding patients who underwent surgery. In total, 1423 hospitals contribute to this database. After the integration of the JBCS database into the NCD, the number of registered breast cancer cases increased significantly. The data are used for planning healthcare services, evaluating the activity of individual surgeons, and for breast cancer research.

Several reports concerning breast cancer in Japan based on data from the JBCS or NCD databases have been published [3–5]. Herein, we provide a summary of the NCD registry data in 2018.

## Patients and methods

The study participants were extracted from NCD data. The inclusion criteria were as follows: patients whose date of surgery was in 2018, and for non-surgery patients, those who began treatment in 2018.

From this database, the demographics, pathological information, and information on surgery, chemotherapy, endocrine therapy, and radiation therapy were extracted. Pathological information was treated according to Japanese and international guidelines [6, 7]. For the purpose of calculating breast cancer cases per 100,000 population, the report “Current Population Estimate as of October 1, 2018” issued by the Statistics Bureau of Japan was used [8]. Estrogen receptor (ER) and progesterone receptor (PgR) were defined as positive when they were expressed at ≥ 1% in the tumor tissue. Human epidermal growth factor receptor type 2 (HER2) positivity was defined according to the 2013 ASCO/CAP guidelines [9].

## Key findings

### Demographics and clinical characteristics of the patients

In 2018, 95,620 breast cancer cases were registered. According to the national registry gathered through local government, 94,519 breast cancer cases were registered [1]. It is not too much to say that almost all of the Japanese breast cancer patients were registered in the NCD database.

Among 95,620 patients with breast cancer, 94,999 patients (99.4%) were women and 621 patients (0.7%) were men. Herein, we studied 94,999 female breast cancer patients (Table 1). Male breast cancer patients were not analyzed in this report.

**Table 1:** Demographics and clinical characteristics of the patients

		Number of patients	Percentage of patients (%)
Number of female patients		94,999	
Laterality	Unilateral	84,964	89.4
	Synchronous bilateral	6265	6.6
	Metachronous bilateral	3770	4.0
Family history^a^	Yes	14,776	15.6
	No	73,235	77.1
	Missing	6988	7.4
Menopausal status	Premenopausal	29,365	30.9
	Postmenopausal	62,833	66.1
	Unknown^b^	2801	3.0
Body mass index	< 18.5	9131	9.6
	18.5–24.9	58,609	61.7
	25.0–29.9	19,371	20.4
	30–34.9	4791	5.0
	35–39.9	1005	1.1
	≥ 40	217	0.2
	Missing	1875	2.0
Detection method	Symptoms	48,189	50.7
	Screening with symptoms	5688	6.0
	Screening without symptoms	26,793	28.2
	Other	13,544	14.3
	Missing	785	0.8

^a^If a patient had first- or second-degree relatives with breast cancer, she was considered to have a family history of breast cancer

^b^Patients with hysterectomy were included in this category

**Fig. 1:**
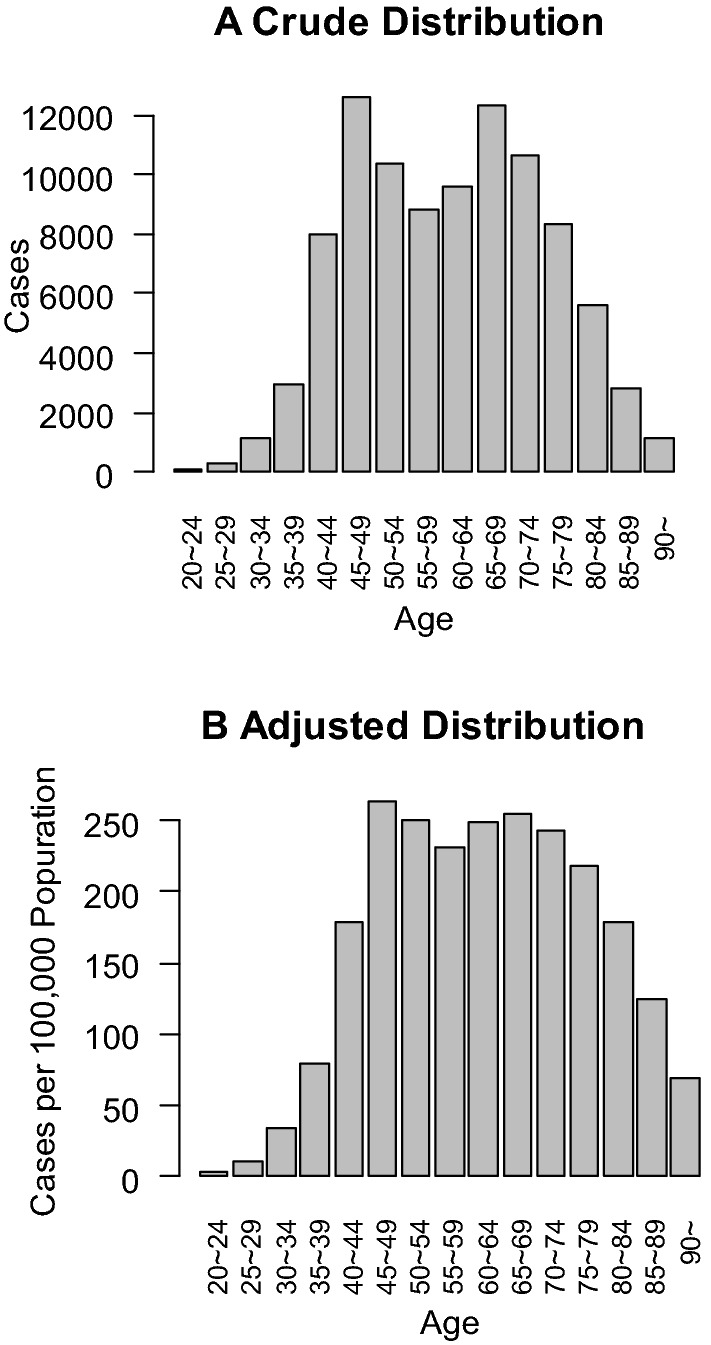
The crude age distribution and the age distribution adjusted per 100,000 population are shown in **A** and **B**, respectively. The age in this database was defined as follows. In patients with preoperative treatment, the age at which they received preoperative treatment was adopted. Those who had no surgical treatment conform with this rule. When patients underwent up-front surgery, the age at which they had surgical treatment was adopted

The median age of the female patients was 65 years. It has been reported that the age distribution of Japanese breast cancer patients is biphasic [3–5]. We confirmed this pattern in the cases registered in 2018, which showed peaks at ages 45–49 years and 65–69 years (Fig. 1A). In addition to this, we created a diagram of the age distribution of the patients per 100,000 population, because Japan had birth surges after World War II (Fig. 1B). Although the distribution curve was still biphasic after adjusting for population, this adjustment reduced its biphasic character.

**Fig. 2:**
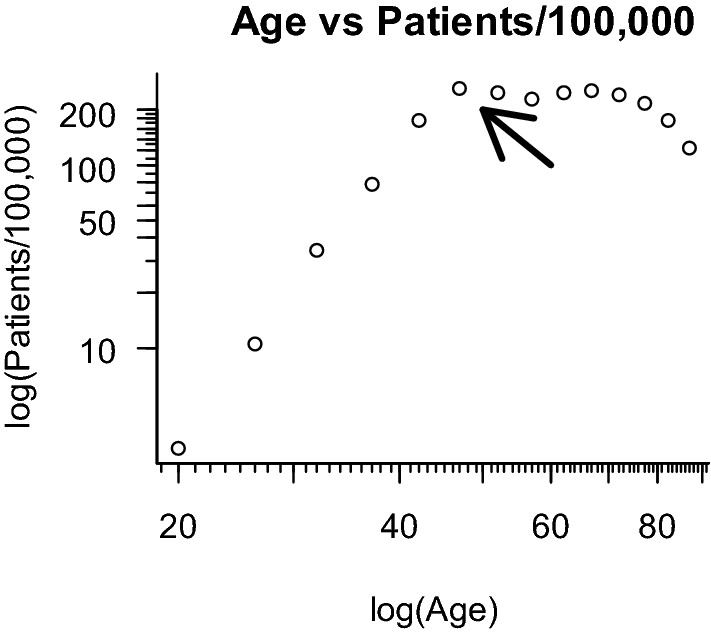
The log–log graph of age versus frequency. The arrow indicates the bend around 50 years of age, which is termed “Clemmesen’s hook”

This biphasic distribution is not specific to Japanese breast cancer. This characteristic has also been reported in the USA and other countries [10]. When cancer develops according to the multistep theory, a log–log graph of age-specific cancer frequency versus age is linear [11]. However, the log–log graph for breast cancer shows a bend around the age of 50 years. This bend is called “Clemmesen’s hook” [12], and was reproduced in our analysis (Fig. 2). Several hypotheses have been suggested to explain Clemmesen’s hook; one is that estrogen plays a role in carcinogenesis [12]. Another is that breast cancer consists of early-onset and late-onset subtypes [13]. However, a definitive explanation remains to be found.

Laterality, family history of breast cancer and menopausal status are shown in Table 1. Synchronous and metachronous bilateral breast cancer accounted for 6.6 and 4.0% of all Japanese breast cancer cases in 2018, respectively. On the other hand, synchronous bilateral breast cancer accounted for only 2.9% of breast cancer patients registered in the Surveillance Epidemiology and End Result (SEER) database in 2014 [14], and other studies report values from 1.0 to 1.5% [15–17]. Although synchronous bilateral breast cancer in Japan looks more frequent, we consider that this frequency is comparable with that in other regions. That is because our annual report defines one unilateral breast cancer as one case. Therefore, one synchronous breast cancer patient counts as two cases.

Our data showed that patients who had a first- or second-degree relative with breast cancer accounted for 15.6% of all female breast cancer patients. The same figures from other countries ranged from 10.0 to 26.7% [18–22]. Our figure was relatively low among these studies.

Breast cancer patients with BMI values of less than 18.5 accounted for 9.6% of all patients. According to the National Health and Nutrition Survey, 12.7% of Japanese women aged 20 years or older had a BMI of less than 18.5 [8]. On the other hand, 26.7% of breast cancer patients had a BMI of 25 or greater, whereas 21.9% of Japanese women in the general population had a BMI of 25 or greater. According to these data, breast cancer patients tended to weigh more than the general population. This finding does not suggest that obesity is associated with breast cancer development, because the age distribution of breast cancer patients is different from that of the general population.

Early detection of breast cancer is crucial to decrease breast cancer death. However, most breast cancers (56.7%) were detected because the patients experienced symptoms or underwent screening after experiencing symptoms. Only 28.2% of breast cancer patients were detected by screening without symptoms. Although the positive impact of breast screening is established [23], education about self-examination of the breast is also important to reduce breast cancer mortality.

The prevalence of comorbidities is shown in Table 2. No comorbidity was reported in 65.7% of patients. The most frequent comorbidity was hypertension (23.4%). Diabetes mellitus, ischemic heart disease, and heart failure were reported in 7.6, 1.9 and 0.9% of patients respectively. These findings are important when anthracyclines and trastuzumab are administered in adjuvant therapy.

**Table 2:** Prevalence of comorbidities

	Number of patients	Percentage of patients (%)
Hypertension^a^	22,187	23.4
Diabetes mellitus^b^	7261	7.6
Malignant neoplastic disease other than breast cancer	5152	5.4
Cerebral or peripheral vascular disease	3037	3.2
Ischemic heart disease	1780	1.9
Renal dysfunction^c^	1463	1.5
Chronic hepatitis	1361	1.4
Collagen disease	1012	1.1
Heart failure	816	0.9
Chronic obstructive pulmonary disease	413	0.4
No comorbidity	62,406	65.7%

^a^Patients were defined as having hypertension if they received anti-hypertension agents

^b^Patients were defined as having diabetes if they received insulin treatment

^c^Patients were defined as having renal dysfunction if their serum creatinine level was over 1.0 mg/dl or if their estimated glomerular filtration rate (eGFR) was less than 60 ml/min/1.73m^2^

### Pathology

**Fig. 3:**
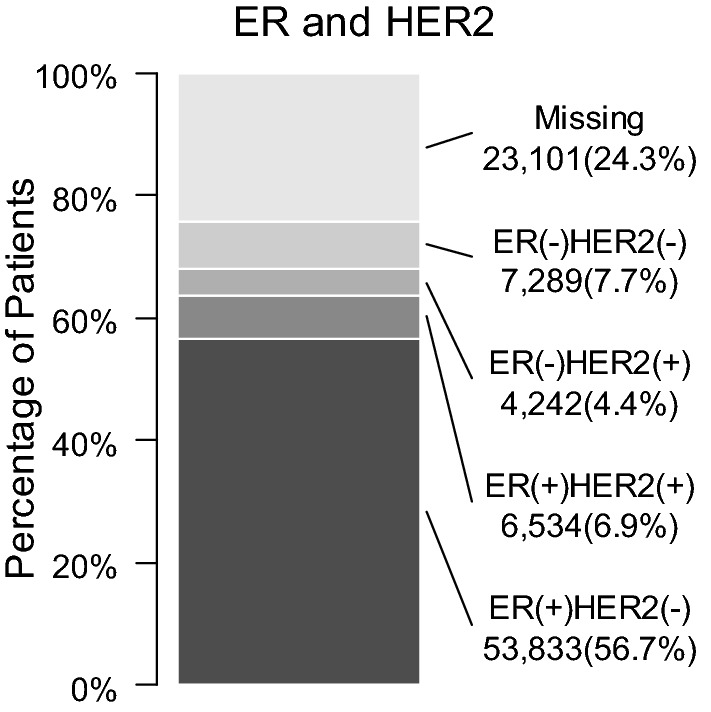
Distribution of ER and HER2. ER and HER2 denote the estrogen receptor and the human epidermal growth factor receptor type 2, respectively

The pathological characteristics based on the tumor, node, metastasis (TNM) classification are summarized in Table 3. Breast cancers classified as Tis or T1 in the tumor factor, N0 in the nodal factor and stage 0 to IIB accounted for 60.6, 81.1 and 88.0%, respectively. Many newly diagnosed breast cancers were at the early stage. The distribution of estrogen receptor (ER) expression and human epidermal growth factor receptor 2 (HER2) expression is presented in Tables 4, 5 and Fig. 3. ER and HER2 were determined based on surgical material. The characterization of ER or HER2 was missing in 24.3% of patients. It is regrettable that this percentage was higher than the value of 7% observed in SEER [24]. Distribution of histological data is shown in Table 6. Histological classification is defined by the committee belonging to Japanese Breast Cancer Society [7].

**Table 3:** Pathological Characteristics

	Number of patients	Percentage of patients (%)
T factor		
Tis	13,600	14.3
T1	43,970	46.3
T2	27,484	28.9
T3	2949	3.1
T4	4648	4.9
Missing	2348	2.5
N factor		
N0	77,021	81.1
N1	12,137	12.8
N2	1972	2.1
N3	1905	2.0
Missing	1964	2.1
M factor		
M0	90,699	95.5
M1	2021	2.1
Missing	2279	2.4
Stage		
0	13,515	14.2
I	40,661	42.8
IIA	22,050	23.2
IIB	7374	7.8
IIIA	2177	2.3
IIIB	3051	3.2
IIIC	1326	1.4
IV	2021	2.1
Missing	2824	3.0

Pathological data were treated according to the Japanese and international guidelines [6, 7]

**Table 4:** Immunohistochemical Characteristics

	Number of patients	Percentage of patients (%)
ER		
Negative	12,902	13.6
1–9%	2751	2.9
10% or more	65247	68.7
Missing	14,099	14.8
PgR		
Negative	21,254	22.4
1–9%	6456	67.8
≥ 10%	53,043	55.8
Missing	14,246	15.0
HER2		
Negative	61,433	64.7
Positive	10,849	11.4
Missing	22,717	23.9

*ER* estrogen receptor, *PgR* progesterone receptor, *HER2* human epidermal growth factor receptor type 2

**Table 5:** HER2 Evaluation According to Immunochemistry and FISH test

	Number of patients	Percentage of patients (%)
HER2 Immunochemistry		
0	25,462	26.8
1 +	27,051	28.5
2 +	14,384	15.1
3 +	8770	9.2
Missing	19,332	20.3
HER2 FISH in HER2 = 2 +		
Positive	2079	14.5
Negative	8920	62.0
Missing	3385	23.5

*FISH* Fluorescence in situ hybridization

**Table 6:** Histology

	Number of patients	Percentage of patients (%)
Epithelial tumors		
Ductal carcinoma in situ	12,903	14.1
Lobular carcinoma in situ	382	0.4
IDC-Papillotubular carcinoma	15,623	17.1
IDC-Solid-tubular carcinoma	12,365	13.5
IDC-Scirrhous carcinoma	27,985	30.6
IDC (not sub-classified)	6472	7.1
Mucinous carcinoma	3228	3.5
Medullary carcinoma	247	0.3
Invasive lobular carcinoma	3938	4.3
Adenoid cystic carcinoma	65	0.1
Squamous cell carcinoma	152	0.2
Spindle cell carcinoma	103	0.1
Apocrine carcinoma	1105	1.2
Tubular carcinoma	362	0.4
Invasive micro-papillary carcinoma	806	0.9
Matrix-producing carcinoma	50	0.1
Other special subtypes	602	0.7
Paget’s disease	250	0.3
Mixed connective tissue and epithelial tumors		
Malignant phyllodes tumor	142	0.2
Carcinosarcoma	7	0.0
Non-epithelial tumors		
Stromal sarcoma	26	0.0
Other non-epithelial tumors	45	0.0
Unclassified tumors	521	0.6
Unknown	3987	4.4

Classification was performed according to the general rules for clinical and pathological recording of breast cancer [7]

*IDC* Invasive ductal carcinoma

### Surgery

**Fig. 4:**
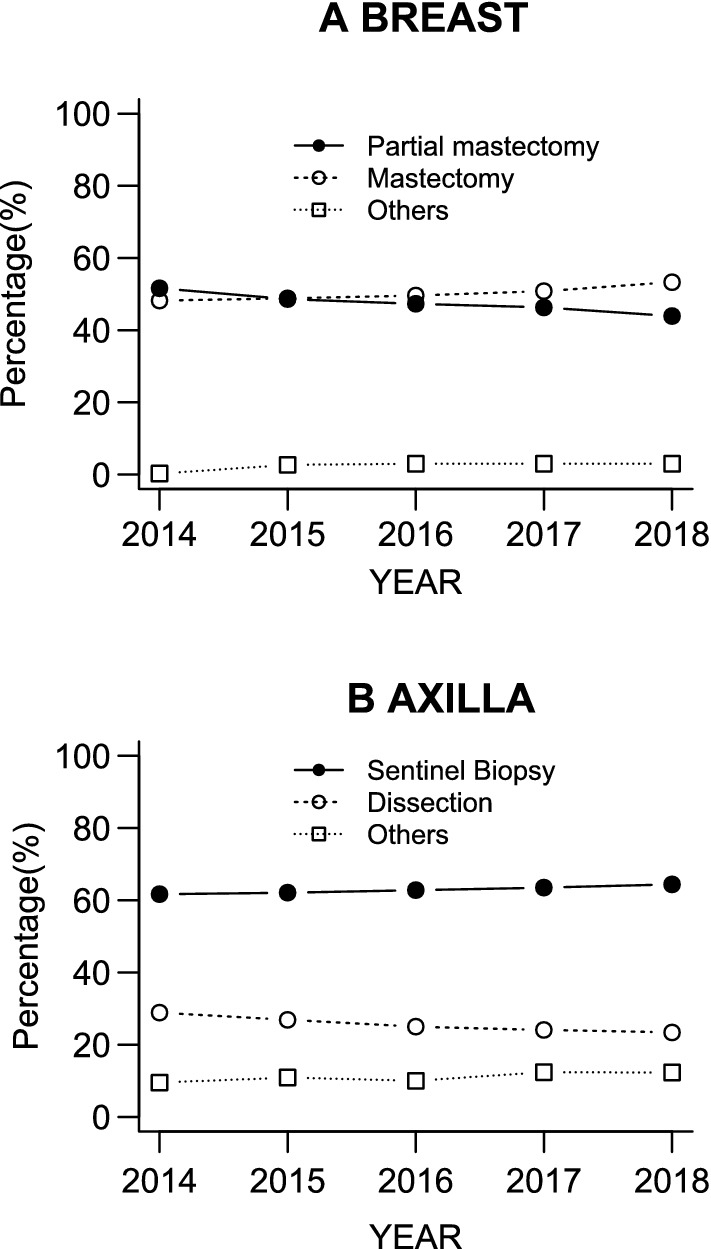
**A** Trends in breast surgery between 2014 and 2018. Mastectomy includes total mastectomy, nipple-sparing mastectomy, skin-sparing mastectomy and radical mastectomy. **B** Trends in axillary surgery between 2014 and 2018. The cases of intraoperative conversion from sentinel node biopsy to axillary dissection are included in the axillary dissection group

The pattern of surgical treatment in patients without distant metastasis is presented in Table 7. Fewer patients underwent partial mastectomy compared to mastectomy, including skin-sparing mastectomy and nipple-sparing mastectomy. Furthermore, the percentage of patients who underwent partial mastectomy decreased over the period 2014–2018 (Fig. 4A). On the other hand, the rate of mastectomy increased over time (Fig. 4A).

**Table 7:** Surgical treatment in patients without distant metastasis

		Number of patients	Percentage of patients (%)
M0 patients with surgery		87,852	
Breast surgery	Partial mastectomy	39,054	44.5
	Mastectomy	46,699	53.1
	Total mastectomy	42,208	48.0
	Nipple-sparing mastectomy	2334	2.7
	Skin-sparing mastectomy	1843	2.1
	Radical mastectomy^a^	314	0.4
	No breast surgery	396	0.5
	Other	1703	1.9
Axillary surgery	No axillary surgery	6804	7.7
	Sentinel node biopsy	57,816	65.8
	Sentinel node biopsy followed by axillary clearance	6915	7.9
	Axillary clearance	13,307	15.1
	Sampling	1192	1.4
	Other	1818	2.1

^a^This procedure means mastectomy with removal of the pectoral muscles

73.7% of patients with surgery had a sentinel node biopsy alone. The number of cases treated with a sentinel node biopsy alone increased slightly over the period 2014–2018 (Fig. 4B). On the other hand, axillary clearance was performed in 23.0% of total patients, including those whose sentinel node biopsy was converted into axillary dissection during the operation.

### Adjuvant systemic treatment

**Fig. 5:**
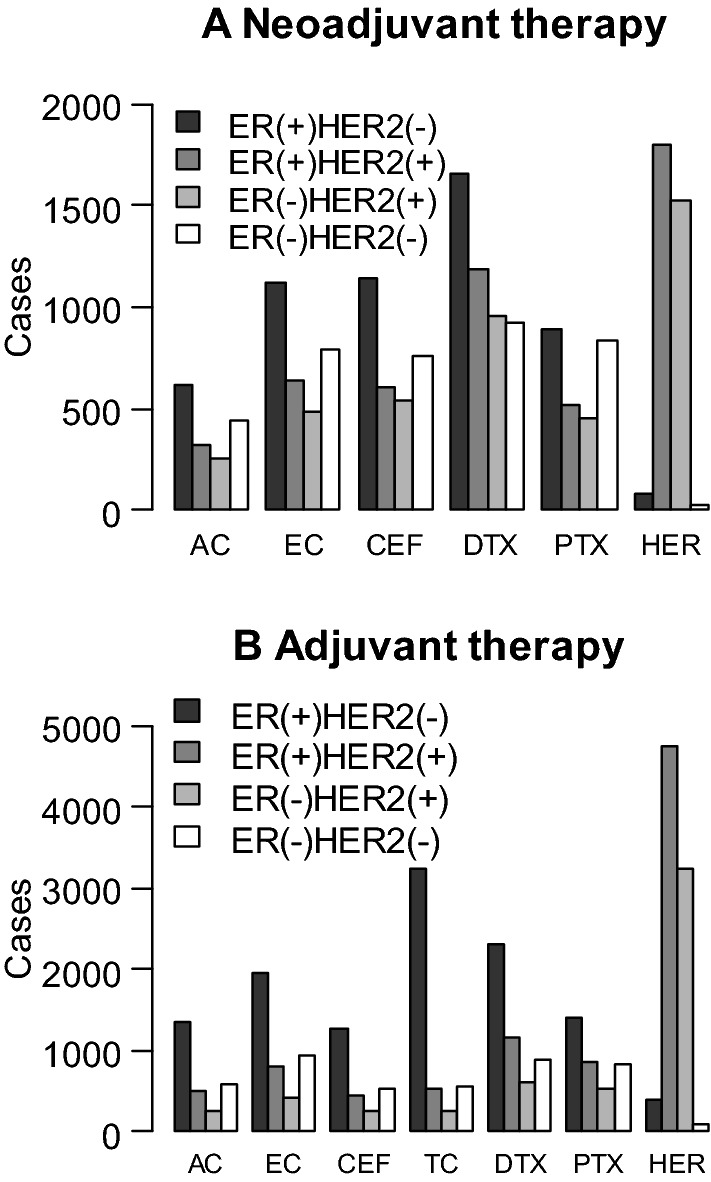
The use of major preoperative (**A**) and postoperative (**B**) chemotherapy regimens stratified by immuno-histological subtype. *AC* Adriamycin and Cyclophosphamide, *EC* Epirubicin and Cyclophosphamide, *CEF* Cyclophosphamide, Epirubicin and Fluorouracil, *DTX* Docetaxel, *PTX* Paclitaxel, *HER* Trastuzumab, *TC* Docetaxel and Cyclophosphamide

A total of 12,846 patients (14.2%) had preoperative treatment (Table 8). Chemotherapy was delivered to 9,551 women (10.5%) and endocrine therapy to 3454 patients (3.8%). Considering the regimens containing anthracyclines, epirubicin was used more frequently than doxorubicin. In the taxane therapy category, docetaxel was given with higher frequency than paclitaxel. Figure 5A demonstrates the preoperative use of major chemotherapy regimens stratified by subtypes. Because there are many ER-positive HER2-negative breast cancer patients, every regimen except for trastuzumab was likely to be used for these patients.

**Table 8:** Preoperative treatment

	Number of patients	Percentage of patients (%)
Preoperative therapy		
Yes	12,846	14.2
Chemotherapy	9551	10.5
Endocrine therapy	3454	3.8
Molecular targeted therapy	3782	4.2
Radiation therapy	82	0.1
Others	160	0.2
No	77,672	85.6
Missing	181	0.2
Total	90,699	
Chemotherapy		
AC or CAF	1711	17.9
EC or CEF	6288	65.8
TC	375	3.9
DTX	4853	50.8
PTX	2782	29.1
nab-PTX	695	7.3
Carboplatin	169	1.8
Others	846	8.9
Total	9551	
Molecular targeted Tx		
Trastuzumab	3523	3.9
Pertuzumab	479	0.5
Bevacizumab	138	0.2
Others	113	0.1
Total	3782	

*AC* doxorubicin cyclophosphamide, *CAF* doxorubicin cyclophosphamide and 5-fluorouracil, *EC* epirubicin cyclophosphamide, *CEF* epirubicin cyclophosphamide and 5-fluorouracil, *TC* docetaxel and cyclophosphamide, *DTX* docetaxel, *PTX* paclitaxel, *nab-PTX* nab-paclitaxel, *Tx* therapy

**Fig. 6:**
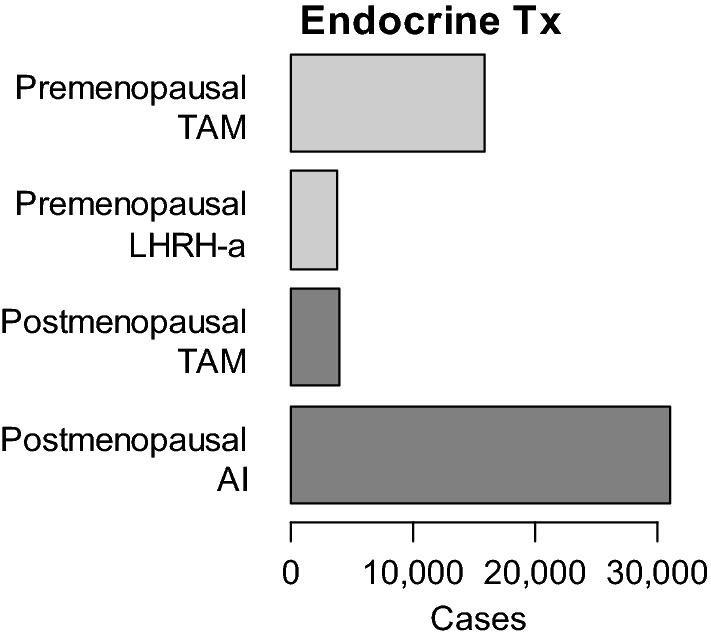
The use of major hormonal agents stratified by menopausal status. *TAM* tamoxifen, *LHRH-a* Gonadotropin-releasing Hormone Agonist, *AI* aromatase inhibitor, *Tx* therapy

A total of 71,278 patients had local or systemic postoperative therapy. As shown in Table 9, chemotherapy was administered to 18,989 women (21.6%), endocrine therapy was given to 54,124 women (61.6%), and radiation therapy was delivered to 35,278 patients (40.2%). In this setting, epirubicin and docetaxel were used more frequently than doxorubicin and paclitaxel, respectively. Figure 5B demonstrates the postoperative use of major chemotherapy regimens stratified by subtypes. TC regimen was likely to be used in ER-positive HER2-negative patients compared with the patients of other subtypes. A total of 33,127 patients received an aromatase inhibitor, 20,426 women received tamoxifen, and 4128 women received gonadotropin-releasing hormone agonist. In this gonadotropin-releasing hormone agonist cases, 3660 cases (88.7%) and 203 cases (5.5%) also received tamoxifen and aromatase inhibitor, respectively. In 157 cases (3.8%), gonadotropin-releasing hormone agonist was used as a single agent. Figure 6 shows the use of major hormonal agents stratified by menopausal status. The ratio of tamoxifen to gonadotropin-releasing hormone was approximately 4 to 1 in premenopausal patients. On the other hand, the proportion of aromatase inhibitor to tamoxifen was around 8 to 1 in postmenopausal patients.

**Table 9:** Postoperative adjuvant therapy

	Number of patients	Percentage of patients (%)
Postoperative therapy		
Yes	71,278	81.1
Chemotherapy	18,989	21.6
Endocrine therapy	54,124	61.6
Molecular targeted therapy	8999	10.2
Radiation therapy	35,278	40.2
Others	1697	1.8
No	12,936	14.7
Missing	3638	4.1
Total	87,852	
Chemotherapy		
AC or CAF	2799	14.7
EC or CEF	7038	37.1
TC	4813	25.3
DTX	5267	27.7
PTX	3740	19.7
nab-PTX	356	1.9
CMF	218	1.1
Carboplatin	892	0.6
Others	1725	9.1
Total	18,989	
Endocrine therapy		
Tamoxifen	20,426	37.7
Gonadotropin-releasing Hormone Agonist	4128	7.6
Aromatase Inhibitor	33,127	61.2
Others	1099	2.0
Total	54,124	
Molecular targeted Tx		
Trastuzumab	8826	98.1
Pertuzumab	1202	13.4
Trastuzumab Emtansine	11	0.1
Others	179	2.0
Total	8999	

With regard to the abbreviated names, refer to the footnote of Table 6

**Fig. 7:**
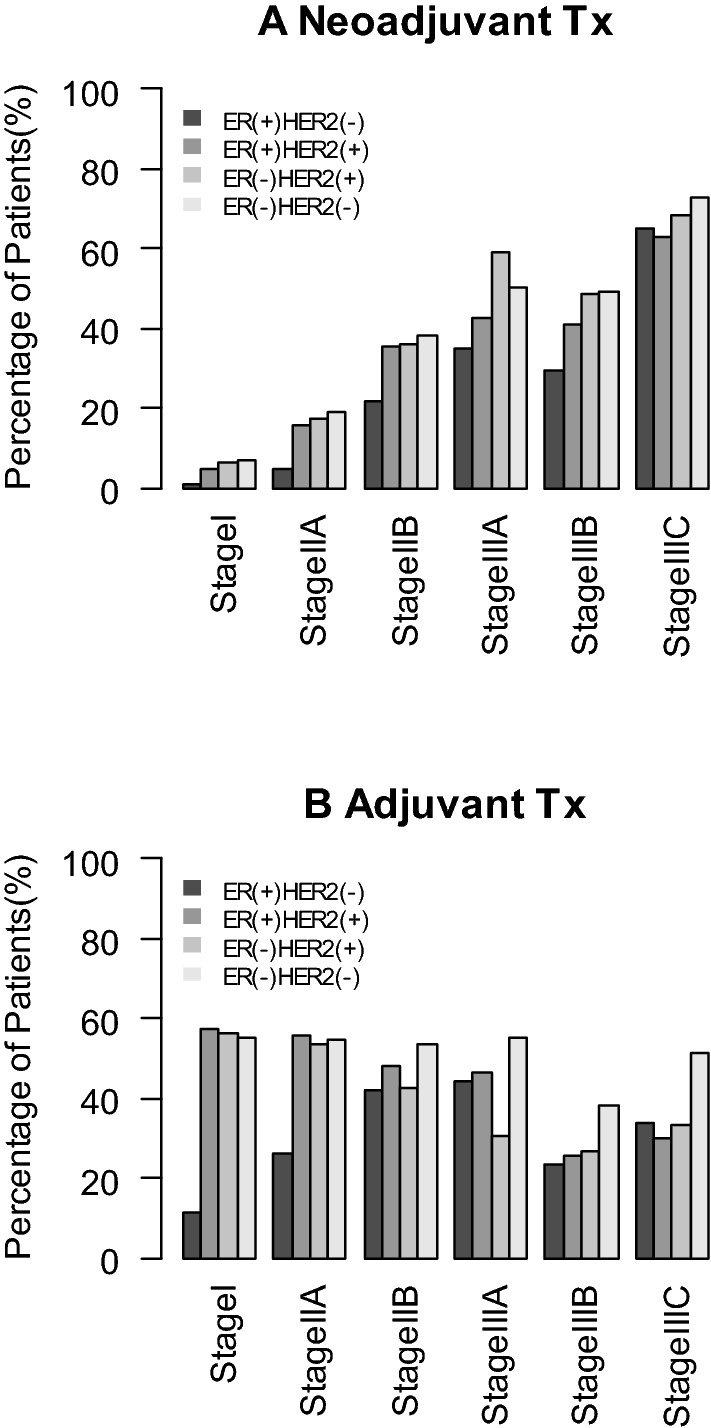
Distribution of patients who received preoperative (**A**) and postoperative (**B**) chemotherapy stratified by stage and immuno-biological subtype. *Tx* therapy

The rate of neoadjuvant and adjuvant chemotherapy administration stratified by stage and receptor expression profile is shown in Fig. 7A and Fig. 7B, respectively. Patients with a higher stage were more likely to receive neoadjuvant chemotherapy. On the other hand, patients with a lower stage are likely to receive adjuvant chemotherapy. Those with Stage I or IIa whose subtype was ER (+) and HER2 (−) tended to do without chemotherapy.

### Radiation therapy

Postoperative radiation therapy among M0 patients is summarized in Table 10. In the partial mastectomy patients, 29,012 patients (74.3%) received radiation therapy. Among these patients with radiation therapy, 27,797 patients (95.8%) and 6,301 patients (21.7%) had radiation at the total breast and boost, respectively. In the total mastectomy patients, 6,166 patients (17.1%) received radiation therapy. Among these patients with radiation therapy, 4969 patients (80.6%) and 4,708 patients (76.4%) had radiation at the chest wall and supra-clavicular region, respectively. Furthermore, 6068 patients had radiation therapy for the supra-clavicular lymph nodes regardless of the radiation to the breast. Considering that at least 4794 patients had 4 or more positive axillary lymph nodes (data not shown), radiation therapy for this region is considered to have been delivered properly.

**Table 10:** Postoperative radiation therapy in M0 breast cancer

	Number of patients	Percentage of patients (%)	Number of patients	Percentage of patients (%)
Partial mastectomy	39,054	100		
Partial mastectomy with radiation	29,012	74.3		
Partial mastectomy with radiation			29,012	(100)
Total breast			27,797	(95.8)
Boost			6,301	(21.7)
Partial breast			967	(3.3)
Supra-clavicular			1,360	(4.7)
Axilla			803	(2.8)
Total mastectomy^a^	42,208	100		
Total mastectomy with radiation	6166	17.1		
Total mastectomy with radiation			6,166	(100)
Chest wall			4,969	(80.6)
Supra-clavicular			4,708	(76.4)
Para-sternal			911	(14.8)
Axilla			848	(13.8)

^a^Total mastectomy includes nipple-sparing mastectomy, skin-sparing mastectomy and radical mastectomy

## Postscript

A total of 94,999 female breast cancer cases were studied. The distribution of patients’ ages was biphasic, which was also observed in other countries. The breast cancer cases detected by screening without symptoms accounted for only 28.2%. Mastectomy was performed more often than partial mastectomy. Epirubicin-containing regimens and docetaxel were used more than doxorubucin-containing regimens and paclitaxel. A total of 74.3% of women with partial mastectomy received radiation therapy, whereas 17.1% of patients with total mastectomy had radiation therapy.

The primary aim of this article is the global announcement of the latest statistics of breast cancer in Japan. Compared with previous reports of NCD breast cancer database, this article addresses some special issues: interpretation of the age distribution, the distribution of cases according to histopathological classification, comorbidities in patients, and practical use of anticancer agents and radiation therapy [3–5].

We defined one unilateral breast cancer as one case. Therefore, one synchronous breast cancer patient counts as two cases. Because synchronous breast cancer cases account for only 6.6% in the NCD database, we believe these cases will not overturn our interpretations of the findings. However, we propose to reinvent format of the future annual reports.

ER and HER2 were determined based on surgical material. Therefore, it is possible that ER and HER2 in neoadjuvant chemotherapy or non-surgery cases were not properly presented. The high rate of missing data in ER and HER2 profile may be due to this procedure. We need to improve this in future report.

Our report has some limitations. The NCD database was originally established based on surgical cases. Therefore, breast cancer patients who undergo surgery are likely to be enrolled in this database. On the other hand, those without surgery are more likely to be missed. That is why the percentage of stage IV breast cancer patients in the NCD was 2.1%, which is lower than another database based on a nationwide survey [1]. Furthermore, the reliability of this database needs to be increased. This concern arises because the data are entered by many busy physicians, and there are no systematic measures to confirm the accuracy.
